# Endocannabinoid control of glutamate NMDA receptors: the therapeutic potential and consequences of dysfunction

**DOI:** 10.18632/oncotarget.10095

**Published:** 2016-06-15

**Authors:** María Rodríguez-Muñoz, Pilar Sánchez-Blázquez, Manuel Merlos, Javier Garzón-Niño

**Affiliations:** ^1^ Department of Molecular, Cellular and Developmental Neurobiology, Laboratory of Neuropharmacology, Instituto Cajal, Consejo Superior de Investigaciones Científicas (CSIC), Madrid, Spain; ^2^ Drug Discovery & Preclinical Development, Esteve, Barcelona, Spain

**Keywords:** σ1R, HINT1 protein, GPCR-NMDAR coordination, convulsive disorders, mood disorders

## Abstract

Glutamate is probably the most important excitatory neurotransmitter in the brain. The glutamate *N*-methyl-D-aspartate receptor (NMDAR) is a calcium-gated channel that coordinates with G protein-coupled receptors (GPCRs) to establish the efficiency of the synaptic transmission. Cross-regulation between these receptors requires the concerted activity of the histidine triad nucleotide-binding protein 1 (HINT1) and of the sigma receptor type 1 (σ1R). Essential brain functions like learning, memory formation and consolidation, mood and behavioral responses to exogenous stimuli depend on the activity of NMDARs. In this biological context, endocannabinoids are released to retain NMDAR activity within physiological limits. The efficacy of such control depends on HINT1/σ1R assisting in the physical coupling between cannabinoid type 1 receptors (CB1Rs) and NMDARs to dampen their activity. Subsequently, the calcium-regulated HINT1/σ1R protein tandem uncouples CB1Rs to prevent NMDAR hypofunction. Thus, early recruitment or a disproportionate cannabinoid induced response can bring about excess dampening of NMDAR activity, impeding its adequate integration with GPCR signaling. Alternatively, this control circuit can apparently be overridden in situations where bursts of NMDAR overactivity provoke convulsive syndromes. In this review we will discuss the possible relevance of the HINT1/σ1R tandem and its use by endocannabinoids to diminish NMDAR activity and their implications in psychosis/schizophrenia, as well as in NMDAR-mediated convulsive episodes.

## INTRODUCTION

The glutamatergic system plays an essential role in neural signaling and as such, the ionotropic *N*-methyl-D-aspartate receptors (NMDARs) influence the operative tone of the synapse by determining the weight assigned to the incoming signals. Unfortunately, a series of neurological disorders concur with dysfunctions of these glutamatergic receptors, such as those produced by the excitotoxicity resulting from their excess activity. As chronic blockade of NMDARs compromises cell viability other approaches must be considered to safely diminish their activity. Accordingly, the regulation of NMDARs by certain G protein-coupled receptors (GPCRs) provides one such therapeutic opportunity. GPCRs and glutamate NMDARs stimulate complex cellular signaling pathways, yet they also exert a mutual regulation on each other's signaling [1, 2]. In this context, the endocannabinoid system, though the activation of the cannabinoid 1 receptor (CB1R), plays a very relevant role in reducing NMDAR activity [3, 4]. Thus, this endogenous system could be pharmacologically manipulated to re-establish the function of dysregulated NMDARs.

There are several excellent reviews on glutamate [5, 6] and endocannabinoids [7, 8] that will bring the reader up to date on what is currently known about these systems. Recent studies have described how the tandem histidine triad nucleotide-binding protein 1 (HINT1) and the sigma receptor type 1 (σ1R) promote physical coupling and uncoupling between the CB1R and the NR1 subunit of the NMDAR [9, 10]. Thus, this review will analyze the negative control that endocannabinoids exert on NMDAR activity and its potential to reduce the incidence of convulsive syndromes like epilepsy, which are mediated by NMDAR hyperactivity, as well as their possible role in provoking NMDAR hypofunction, such as that accompanying psychosis/schizophrenia.

## I THE GLUTAMATE NMDA RECEPTOR

Glutamate is the major excitatory neurotransmitter in the CNS [11], and it activates both ionotropic and metabotropic receptors. Ionotropic receptors directly gate ion passage and they are divided into three major subclasses: α-amino-3-hydroxy-5-methylisoxazole-4-propionic acid (AMPA), kainate, and NMDA receptors. Of these, the NMDARs have received much attention because their deregulation is observed in many neurological disorders, such as neurodegenerative diseases [12], neuropathic pain [13, 14], mood disorders and psychosis-schizophrenia [15, 16].

The NMDAR is a ligand-gated cation channel that is highly permeable to monovalent ions and Ca^2+^. Binding of glutamate opens the channel pore, and the concurrent binding of glycine increases the amplitude and time course of ion flux. NMDARs are composed of NR1, NR2 (A, B, C and D) and NR3 (A and B) subunits, and the functional NMDAR is a tetramer consisting of a pair of NR1 subunits each associated to at least one type of the NR2/3 subunits [17]. NMDAR activation enhances the binding of cytosolic Ca^2+^ to calmodulin (CaM), propagating this signal through many other proteins, including kinases (*e.g*., CaMKII), phosphatases (*e.g*., calcineurin and serine/threonine protein phosphatase 1 -PP1), neural nitric oxide synthase (nNOS) and adenylyl cyclase (types I, III and VI) [18, 19].

## II CROSS-REGULATION BETWEEN GPCRs AND NMDARs

The NMDAR is essential for neuronal plasticity and differentiation, brain development and synaptic plasticity, directly affecting learning and memory consolidation [20]. Temporal and/or spatial coincidence determines the weight that a neural cell assigns to the incoming signals, and this weight is influenced by the degree of excitability that glutamate NMDARs confer to the post-synapse. However, the activity of NMDARs also falls under the influence of GPCRs and for example, the acetylcholine type 1 muscarinic receptor dampens NMDAR function *via* the activation of tyrosine phosphatases [21]. In addition, the serotonin 5HT1A [22], adrenergic α1 and α2 [23] and group III mGluR7 receptors [24] impair NMDAR turnover, while other receptors like the CB1R can promote the co-internalization of NMDAR NR1 subunits [25]. Other GPCRs exert the opposite effect, enhancing NMDAR calcium flux via Gβγ/PLCβ/PKC signaling and the non-receptor tyrosine kinase Src [26, 1], including the mu-opioid receptor (MOR) [27], the dopamine D1 receptor [28], group I metabotropic glutamate receptors (mGluR1/5), group II mGluR2/3 [29, 30], and the serotonin 5HT2A/C receptor [31]. Accordingly, the activity of neural cells is influenced by the complex array of signals that are tightly integrated, harmonizing GPCR-triggered signaling cascades and NMDAR glutamate responses. For example, MOR activation recruits NMDARs, exerting a negative influence on opioid signaling by restraining their capacity to produce analgesia, thereby contributing to the development of tolerance [32, 33]. Similarly, NMDAR activity provokes endocannabinoid release and cannabinoid receptor stimulation, in turn diminishing NMDAR activity and preventing excitotoxicity [34].

An interaction that has generated significant interest of late is that between GPCRs and NMDARs during the dynamic process that supports their cross-regulation [2]. The C terminus of NMDAR NR1 subunits is composed of C0-C2(C2′) or of C0-C1-C2(C2′) domains, and the NMDAR NR1 subunits that carry the C1 region bind to the C terminus of the dopamine D1 receptor [35], that of group I metabotropic glutamate receptor (mGlu5a) [36], the MOR [37] and the CB1R [25] when studied *in vitro* and in cell assays. Indeed, *ex vivo* assays performed on different areas of the mouse brain show that these GPCRs co-precipitate with NMDAR NR1 subunits [37, 38, 25]. Moreover, the physiological relevance of the complexes containing MOR/CB1R-NMDAR NR1 subunits is confirmed by their dynamic arrangement under the control of the HINT1 and σ1R [9, 39].

## III THE GPCR-NMDAR CONNECTION: THE HINT1-σ1R TANDEM

At the neural plasma membrane, the HINT1 protein forms complexes with cytosolic regions of different GPCRs [40]. In this environment HINT1 serves as a scaffold for signaling proteins that work together to couple GPCR activity with that of glutamate NMDARs. Among the proteins that HINT1 associates with are protein kinases like PKCγ and PKCα [41], and proteins of the Rz subfamily “Regulators of G-protein signaling” (RGS), mostly RGSZ1(20) [42]. These RGS-Rz proteins have a zinc-finger in their N terminal sequence [40] and they bind to the N terminal PDZ domain of nNOS. HINT1 also connects the Raf-1/MEK/ERK1-2 cassette to GPCRs and the NMDAR NR1 subunits that carry the C1 segment [43]. Significantly, the docking of proteins to HINT1 is organized by Redox signaling, zinc metabolism and PKC activity [33].

**Figure 1:**
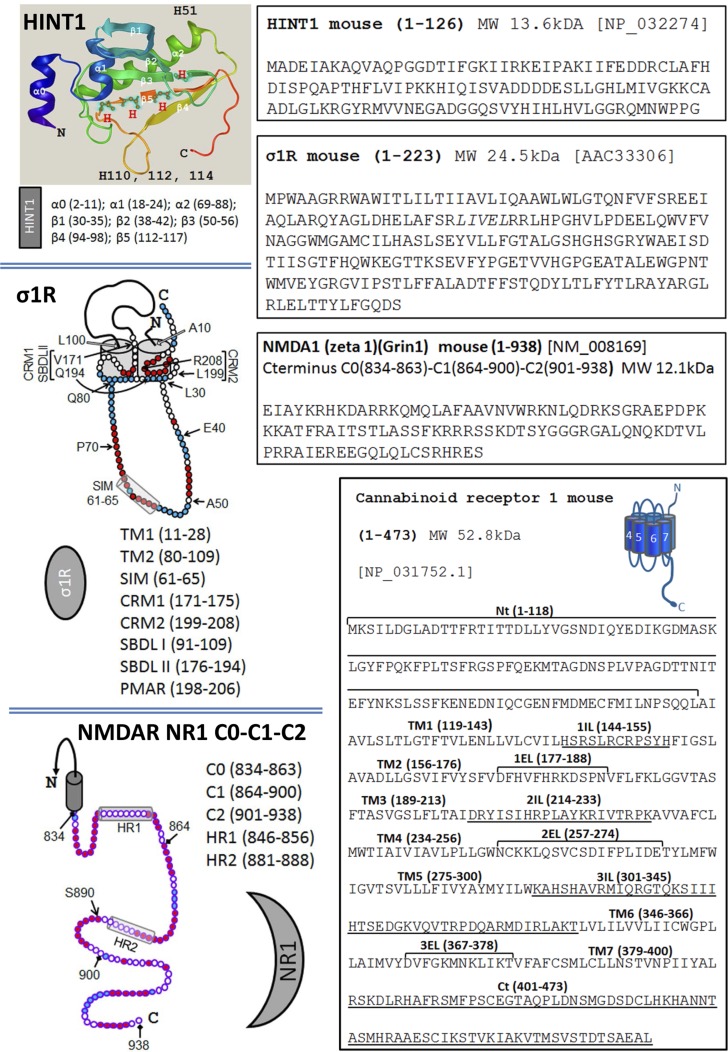
The sequence of HINT1, σ1R, the C terminal of the NMDAR NR1 subunit and the CB1 receptor Sequence of the murine HINT1 protein. The α and β regions, and the histidines are indicated on the ribbon backbone (Novafold/Protean 3D/DNASTAR v12). The long isoform of the murine σ1R has two hydrophobic transmembrane regions, TM1 and TM2. The σ1R hairpin loop (L30-Q80) contains a SUMO-Interacting motif (SIM: 61-65), while the C-terminal region includes two cholesterol-binding motifs, CRM1 and CRM2, and a potential membrane attachment region (PMAR). The steroid binding site is formed by the SBDL I in TM2 and SBDL II at the C terminus [49, 62, 9]. The C terminal C0-C1-C2 of the NMDAR NR1 subunit contains 104 residues with two hydrophobic regions HR1 and HR2 [9]. The S890 residue is indicated, a PKC regulatory site. In the sequence of the murine CB1R, the extracellular, transmembrane and cytosolic regions are indicated.

The σ1R is a linear protein that is widely expressed in nervous tissue [44] and that was initially considered as a type of opioid receptor [45]. However, its amino acid sequence has no significant homology with any other mammalian protein, and it lacks glycosylation sites and a known transducer system [46]. The σ1R interacts with lipid membranes and in the absence of third party proteins this receptor can form oligomers *in vitro*, probably trimers, with each monomer anchored to the lipid membrane by its N terminal region [47]. In the ER and plasma membrane, the σ1R associates with different signaling proteins and in these interactions it apparently displays two transmembrane domains, adopting different conformations [48, 49, 50, 51]. Thus, the σ1R N and C termini are either cytoplasmic [51, 52], or in the context of its interaction with NMDAR NR1 subunits, both the N and C terminal sequences project into the extracellular space through two transmembrane domains [48, 9] (Figure 1). The σ1R does not fulfill the criteria of a typical membrane receptor but its associations with other signaling proteins may be altered through a series of endogenous and exogenous substances, as well as by calcium [49, 53, 10]. Thus, the molecular structure of the σ1R and its different arrangements suggests it fulfills different functions, most likely that of a ligand-regulated chaperone [46].

The pharmacology of the σ1R is complex, with exogenous ligands showing different profiles depending on the system under study [54]. Thus, σ1R ligands influence NMDAR function *in vivo* and *in vitro* [61, 39], and σ1Rs bind to other proteins in the endoplasmic reticulum and plasma membrane in a calcium-dependent manner in cellular expression systems and *in vitro* assays, NMDARs included [9, 49, 62]. Nevertheless, σ1R ligands are therapeutically interesting to treat neurological diseases [55], substance abuse syndromes [56], and NMDAR-related neural dysfunctions (such as certain neuropsychiatric disorders [53], and the allodynia and hyperalgesia that accompanies neuropathy in different animal models [57, 58], as well as potentially serving as adjuvants of opioid analgesia [59, 60].

**Figure 2:**
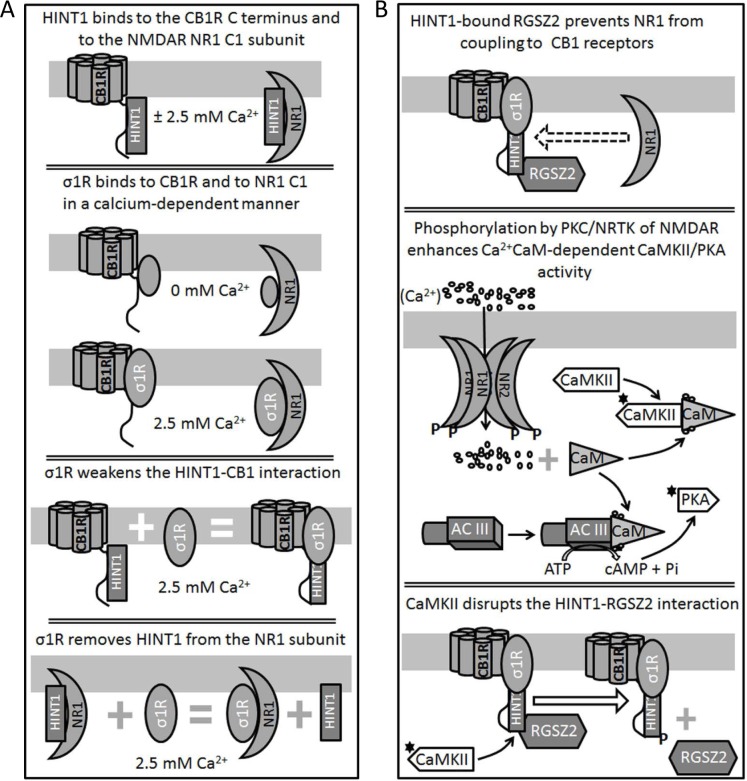
Diagram showing the relationship between HINT1 proteins and σ1Rs in their association with the NMDAR NR1 C1 subunits and CB1Rs **A.** Binding of HINT1 and σ1R to NR1 subunits and CB1Rs. Their interaction is unidirectional and while HINT1 does not dampen σ1R binding to CB1R or NR1 subunits, the σ1R dissociates HINT1 from the NR1 and weakens its interaction with the CB1R. **B.** CB1Rs bind to the NMDAR NR1 subunits via HINT1 proteins. The binding of RGS-Rz proteins to HINT1 prevents the formation of the CB1R-NMDAR complex and, NMDAR-activated CaMKII removes this barrier to make their coupling and cross-regulation possible.

The activity of σ1R is coordinated with that of HINT1 to connect GPCRs with NMDARs and promote (*e.g*., MOR) or reduce (*e.g*., CB1R) its glutamatergic activity [9, 25, 3]. A series of molecular studies have shed some light on how this molecular switch brings NMDARs under the control of GPCRs. Whilst, HINT1 binds to cytosolic sequences of GPCRs and of NMDAR NR1 subunits in a calcium-independent fashion, the association of the σ1R with these signaling proteins increases greatly in the presence of physiological levels of calcium (*i.e*.: low mM range). The relationship between both proteins is asymmetric, and whilst the σ1R prevents HINT1 binding to NR1 subunits and it weakens the association of HINT1 with GPCRs, neurosteroids but not HINT1 alter σ1R binding to these proteins [39, 10] (Figures 1 & 2A). In this environment, high calcium and σ1R agonists such as pregnenolone sulfate enhance the association of σ1Rs with the NR1 C1 subunits, whilst they diminish the binding of σ1Rs to GPCRs, consequently strengthening that of HINT1 to GPCRs. Thus, σ1R agonists restrain the control of GPCR-HINT1 complexes to NR1 subunits that are free of σ1Rs, *e.g*., silent or weakly active NMDARs. In these circumstances, the presence of the σ1R at the GPCR prevents the transfer of HINT1 from the GPCR to the NMDAR [49, 62, 9]. Conversely, regulation by σ1R antagonists like progesterone differs from that of agonists, and whilst antagonists do not alter or only slightly diminish the binding of σ1Rs to GPCRs, weakening the GPCR-HINT1 association, they do drive the removal of σ1Rs from activated NMDARs and they promote the transfer of HINT1 proteins from GPCRs to NR1 C1 subunits [39, 10]. As a result, NMDARs are uncoupled from the influence of GPCRs, be it positive or negative. These observations indicate that the HINT1-σ1R tandem is physiologically driven by calcium and the putative endogenous ligands of σ1Rs are neurosteroids.

## IV CROSS-REGULATION BETWEEN NMDARs AND CB1Rs

### IV.1 Molecular aspects

The HINT1-σ1R protein tandem highlighted above works as a flip-flop switch connecting and disconnecting the activity of GPCRs with that of NMDARs carrying the C1 cytosolic segment within the NR1 subunits, and it can enhance (*e.g*., MOR) or dampen (*e.g*., CB1R) glutamate signaling. As part of this molecular switch, HINT1 physically connects the GPCR to the NMDAR, the ON situation, and when it moves from the GPCR towards the NMDAR it uncouples both receptors, the OFF state. In the GPCR environment, the σ1R weakens the HINT1-NR1 association and it is crucial to maintain the HINT1 protein bound to the GPCR. Thus, its physiological or pharmacological removal brings about HINT1 transfer to the NMDAR NR1 subunit [10, 39]. Following the formation of the GPCR-HINT1-σ1R-NMDAR complex, the activation of receptors like the MOR increases the activity of the coupled NMDAR *via* PKC/Src. The action of PKC promotes the separation of the MOR-HINT1 complex from the phosphorylated NR1 C1 region that now carries the σ1R. On the other hand, Src phosphorylates tyrosine residues of NR2 subunits and increases calcium permeation, favoring σ1R binding to the NMDAR. Thus, activated and phosphorylated NMDARs display low affinity for the HINT1 protein and this precludes their unproductive coupling to the MOR. This cycle would commence when a σ1R plus a silent NMDAR (unphosphorylated) reach the MOR-HINT1 complex, and it ends with the release of the phosphorylated and active NMDAR [9]. Notably, antagonists impair σ1R binding to NMDARs, even in the presence of high calcium. In these circumstances, and before PKC reaches all its targets on the NR1 C1 segment, HINT1 rather than σ1R switches from the GPCR to this region of the coupled NMDAR. Thus, σ1R antagonists promote the separation of MORs from NMDAR-HINT1 complexes and disrupt the cross-regulation between these receptors. Pharmacologically we can take advantage of σ1R antagonists as adjuvants of opioid antinociception with a view to reducing the development of opioid tolerance [60].

In contrast to what is observed for the MOR, the CB1R hinders the activity of NMDARs. As witnessed for the MOR, the CB1R also forms CB1R-HINT1-σ1R complexes with non-phosphorylated NMDARs [10, 39]. However, there is no activation of the NMDAR in the CB1R environment and the σ1R remains at the GPCR allowing endocannabinoids to stabilize the weak activity of NMDARs. As observed for MORs in their interaction with NMDARs, in the absence of σ1R ligands or in the presence of σ1R agonists, the HINT1-σ1R switch enables CB1Rs to associate with inactive NMDARs, the ON situation. By contrast, σ1R antagonists promote the shift of HINT1 from the CB1R to the NMDAR NR1 subunit disconnecting both receptors, the OFF state, thereby preventing cannabinoids from producing NMDAR hypoactivity [39, 10].

**Figure 3:**
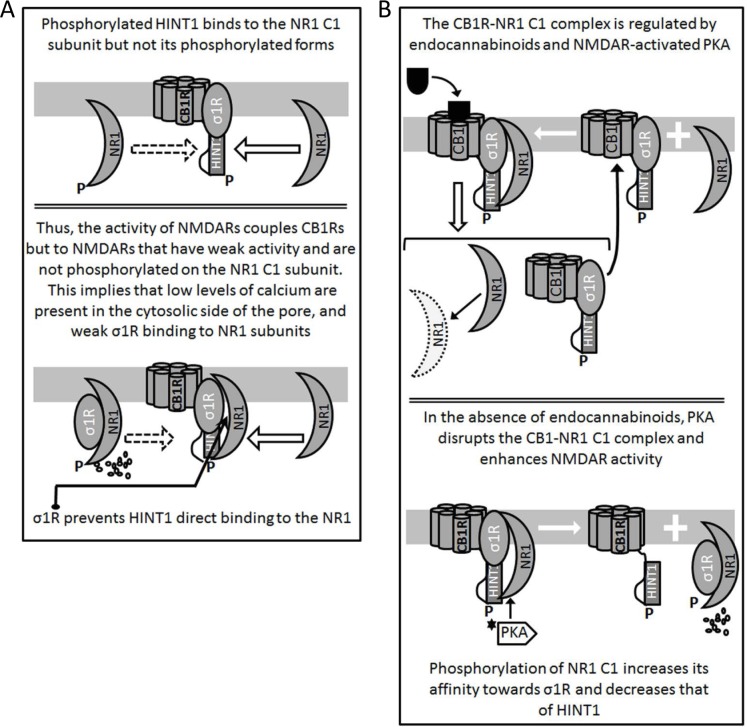
Formation and regulation of the CB1R-NMDAR complex **A.** CB1R-coupled HINT1 proteins freed of RGS-Rz proteins bind to the C1 region of NR1 subunits. PKC/PKA phosphorylation, such as that of activated NMDARs weakens the HINT1-NR1 C1 association (P-S890, 896, 897) while P-T879 abolishes it [9]. **B.** Cannabinoids and PKA determine the fate of the CB1R-NMDAR complex. When cannabinoids bind to CB1Rs they dampen the activity of the coupled NMDARs and they may even provoke the co-internalization of NR1 subunits. As a result, overall NMDAR activity diminishes. However, in the absence of CB1R-bound cannabinoids, PKA acts on the NR1 C1 segment and disrupts its association with HINT1 in the CB1R complex.

In the absence of GPCR or NMDAR activity, binding of GPCR-associated HINT1 proteins to resting NMDARs is blocked by sumoylated RGS-Rz proteins, mostly RGS17 and RGS20 [42, 63, 3]. It is the activity of MOR-activated PKCγ or of NMDAR-activated CaMKII that disrupts the HINT1 interaction with the RGS-Rz barrier [63, 9, 25], thereby allowing the MOR/CB1R-HINT1 complex to associate with the NMDAR NR1 subunits (Figures 2B & 3A). Thus, the HINT1-σ1R tandem sustains the association between these GPCRs and NMDARs, and in the absence of HINT1 proteins or of σ1Rs their relationship is disrupted [37, 9, 39]. Indeed, in HINT1^−/−^ or σ1R^−/−^ mice, the MOR and the NMDAR are physically and functionally uncoupled, and thus, morphine does not recruit NMDARs nor do NMDARs dampen opioid antinociception [63, 9]. Similarly, in these mice cannabinoids fail to reduce NMDAR calcium influx and the subsequent release of endogenous zinc, and they also provide no protection against NMDAR excitotoxicity [25, 4].

### IV.2 Functional aspects

If the activity of NMDARs reaches a given threshold, excitatory signals recruit the negative control of the endocannabinoid system *via* CB1Rs [34]. Thus, the NMDAR-induced release of endocannabinoids [64] provokes the stabilization of CB1R-HINT1 complexes along with silent NMDARs [25, 39], thereby reducing the pool of NMDARs that can be potentially activated (Figure 3A). Since exocannabinoids internalize CB1Rs better than endocannabinoids [38], they promote the co-internalization of the CB1R-HINT1 complexes bound to NR1 subunits and probably, to surface NMDAR NR2 subunits as well [65, 25]. Thus, exocannabinoids better disassemble and inactivate CB1R-associated NMDARs efficiently reducing the risk of the excitotoxicity mediated by NMDAR calcium influx. Notwithstanding, the absence of σ1Rs disrupts the control cannabinoids exert on NMDAR excitatory signaling. In these mice, CB1Rs are separated from the NR1 subunits and the HINT1 proteins switch to the NR1 C1 subunits [9, 39].

## V THE CB1R-NMDAR COMPLEX

### V.1 Functional relevance

Abnormally high spiking activity can damage neurons and the endogenous cannabinoid system provides on-demand protection against acute excitotoxicity. A series of studies suggest that the endocannabinoid system controls NMDAR activity intracellularly through signaling pathways that converge on those triggered by the glutamate receptor [66, 67, 68, 69], although other studies indicate that this control is the result of direct physical coupling between CB1Rs and NMDAR NR1 subunits [25, 39]. In this respect, the absence of CB1Rs abrogates the control that endocannabinoids exert on NMDAR activity, whilst the pharmacological antagonism of NMDARs decreases cannabinoid CB1R mRNA expression [67, 34] [67, 34]. The CB1R is one of the most abundant GPCRs in the nervous system and although it is mostly localized at the pre-synapse, it is also present in the somata and dendrites [70, 71]. Moreover, there is immunocytochemical and ultrastructural evidence that CB1Rs exist in the post-synapse, both at the spinal [72, 68, 73] and supraspinal level [74, 71], co-localizing with NMDARs and PSD95 proteins [75, 25].

The presence of NMDARs in the pre-synapse [76, 77] makes the physical association between CB1Rs and NR1 subunits possible at both sides of the synaptic cleft. As such, pre-synaptic CB1Rs could reduce the release of glutamate into the cleft, contributing to NMDAR hypofunction [78], whereas post-synaptic CB1Rs might interfere with intracellular NMDAR signaling [66], thereby negatively regulating the activity of glutamate by directly inhibiting calcium influx [66, 79]. This latter possibility is also supported by whole-cell patch clamp recordings [78]. Thus, besides interfering with NMDAR signaling, cannabinoids can also directly diminish NMDAR mediated calcium flux channel. In this respect, the control exerted by cannabinoids on NMDAR calcium influx, zinc metabolism and excitotoxicity requires CB1Rs, HINT1 and σ1R proteins. In the absence of the σ1Rs or HINT1 proteins, cannabinoids cannot control NMDARs yet the expression of these proteins in HINT1 and σ1R deficient mice restores the cross-regulation between CB1Rs and NMDARs [4, 9]. These observations bring to the fore the role of HINT1 and σ1R proteins in the restraint that endocannabinoids exert on NMDAR function through CB1Rs.

The HINT1-σ1R machinery couples the CB1R to the NMDAR and controls its capacity to promote oxidative stress, a regulatory event in which PKA plays an essential role. Thus, NMDAR activity augments the formation of the Ca^2+^-CaM that regulates adenylyl cyclase activity, primarily that of types I and VIII but to a lesser extent that of type III, increasing cAMP levels and consequently PKA activity [80, 81]. PKA phosphorylates the protein inhibitor-1 and inhibits the PP1 responsible for dephosphorylating P-Thr286 and inhibiting CaMKII [18, 19]. CaMKII displaces RGS-Rz proteins from HINT1 proteins associated to CB1Rs, an event that is necessary for endocannabinoids to promote and stabilize the inhibitory association of CB1Rs with NMDARs [25] (Figure 3B). Notwithstanding, PKA also favors NMDAR stimulated Ca^2+^ currents, disrupting the CB1R-NMDAR complexes not affected by endocannabinoids, and thereby preserving glutamate function [25] (Figure 3B). Hence, the formation of the CB1R-NMDAR complex requires HINT1 and σ1R but also, endocannabinoids and NMDAR-activated PKA, exerting bidirectional control on this mechanism.

**Figure 4:**
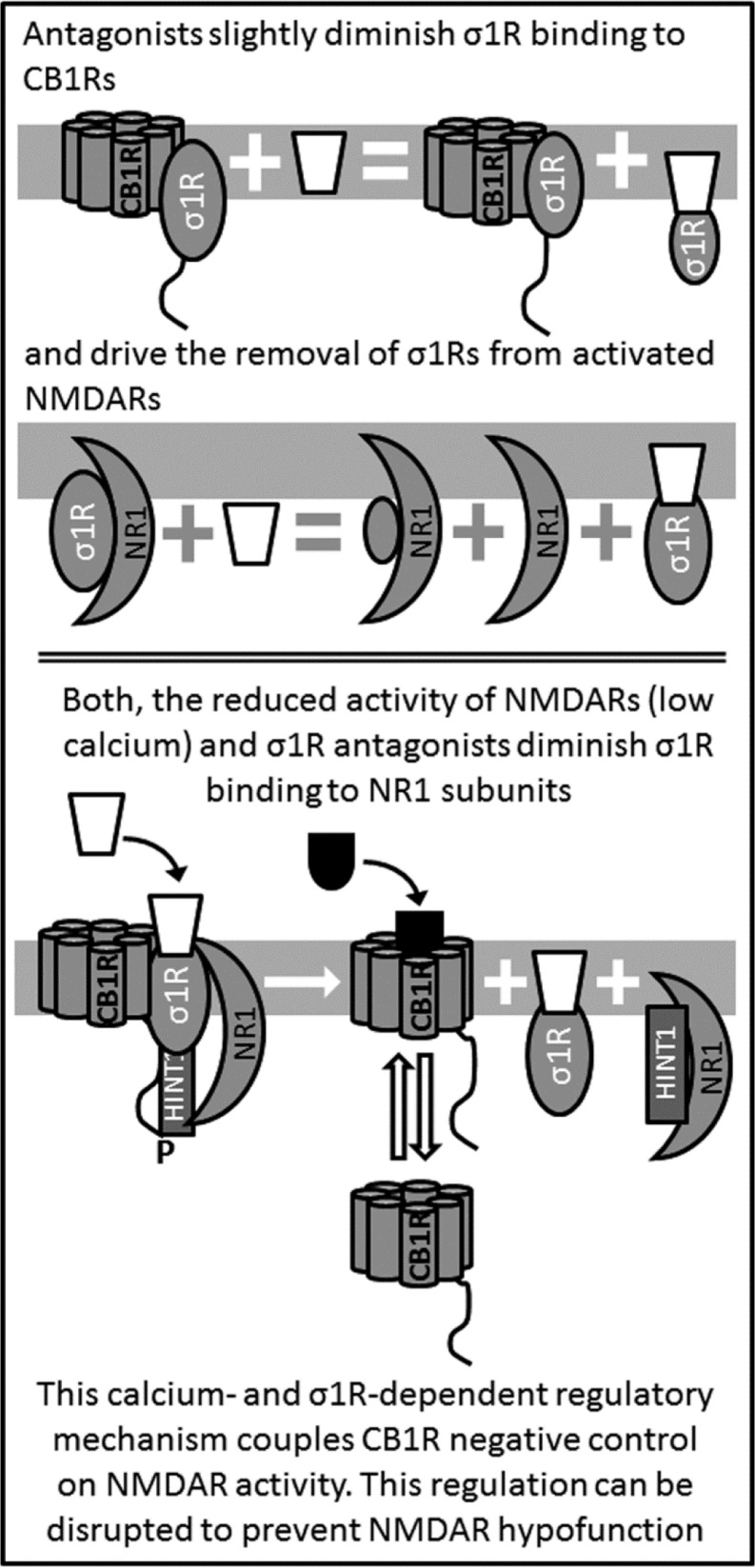
Antagonists of σ1R release NMDAR from the negative control of CB1Rs

When NMDAR activity decreases, the calcium concentration falls, as does the strength of Ca^2+^-CaM/AC/cAMP/PKA/CaMKII signaling. In these circumstances, the formation of CB1R-NMDAR complexes diminishes, and the NMDARs in the existing complexes display little or no activity. Thus, both receptors should be disconnected to prevent undesirable glutamate hypofunction, and in conditions of low calcium/low PKA activity, this regulation is achieved by transferring of HINT1 proteins from CB1Rs to the NMDAR NR1 C1 subunits (Figure 4). The σ1R and its endogenous regulators, probably neurosteroids, apparently play an essential role in this physiological process, which releases NMDAR activity from the negative control of cannabinoids [39, 9].

### V.2 Implications in neural disturbances

Our current understanding of glutamate NMDAR neurotransmission enables us to better define the benefits and risks of its pharmacological manipulation. Whilst, excessive NMDAR activity can be excitotoxic, compromising cell viability, too little activity dysregulates the coordination between GPCRs and NMDARs to set synaptic tone [82]. In order to prevent these anomalies, the endogenous cannabinoid system collaborates with NMDARs to maintain their activity within physiological limits.

#### V.2.1 Psychosis/Schizophrenia - NMDAR hypofunction

The prolific amount of data being generated through studies into psychosis/schizophrenia suggests that both GPCRs and NMDARs participate in the pathophysiology of these mental illnesses. Alterations to GPCRs, like dopamine and GABA receptors, concur with a decrease in NMDAR activity in patients suffering psychosis/schizophrenia [83, 84, 85]. The relationship between these GPCRs and NMDARs is bidirectional, and this cross-regulation could account for the disturbances observed between NMDAR transmission and dopamine receptors in schizophrenia. Indeed, experimental NMDAR hypofunction (induced by its antagonists) causes glutamate metabotropic hyperfunction and dopaminergic hypofunction in the prefrontal cortex, as well as inducing psychotic symptoms and neurocognitive disturbances similar to schizophrenia [83, 16]. Hence, NMDAR dysfunction appears to lie at the crux of the hierarchy of events provoking schizophrenia.

Several clinical and neurobiological findings suggest that endocannabinoids are implicated in NMDAR dysfunction and thus, in the pathophysiology of schizophrenia [86, 87]. The CB1R gene (*CNR1)* maps to chromosome 6q14-15 and linkage studies have suggested a schizophrenia-susceptibility locus lies in this region [88, 89]. A variety of *CNR1* polymorphisms have been studied for associations with schizophrenia, with mixed results [90, 91, 92, 93]. Post-mortem studies carried out on the brains of patients with schizophrenia have demonstrated alterations to the CB1R, such as reduced levels of its mRNA and protein expression in the dorsolateral prefrontal cortex [94], or increased CB1R binding in the corticolimbic areas implicated in this disorder [95, 96, 97]. Accordingly, it is commonly accepted that prolonged cannabis consumption precipitates symptoms of psychosis in vulnerable subjects [98, 99, 100], as well as triggering the relapse of psychotic symptoms in schizophrenic patients and worsening other symptoms of schizophrenia [87, 101].

The endogenous cannabinoid anandamide is involved in regulating pain, mood and cognition [102], and its content in cerebrospinal fluid and plasma augments in patients with schizophrenia, although these levels are negatively correlated with the intensity of the symptoms experienced by these subjects [103, 104, 105]. Pharmacological blockade of anandamide degradation in rodents appears to attenuate certain psychotic-like behaviors induced by amphetamine and phencyclidine [106]. Conversely, the psychotic symptoms induced by Δ^9^-tetrahydrocannabinol and other cannabinoid agonists in healthy volunteers [107, 108] and schizophrenic patients [109] suggest that hyperactivity of the endocannabinoid system contributes to the psychotic state. Thus, it is unclear whether endocannabinoids protect against or intensify schizophrenia [110]. The evidence suggests that exocannabinoids more effectively precipitate psychotic symptoms than endocannabinoids, and that they may even play opposite roles in the expression of this mental illness. The effect of CB1R antagonism in schizophrenia has been evaluated in pre-clinical and clinical studies yielded promising although not definite results [111, 112, 113]. Unfortunately, few studies are available on the therapeutic use of cannabinoids in psychosis and schizophrenia.

**Figure 5:**
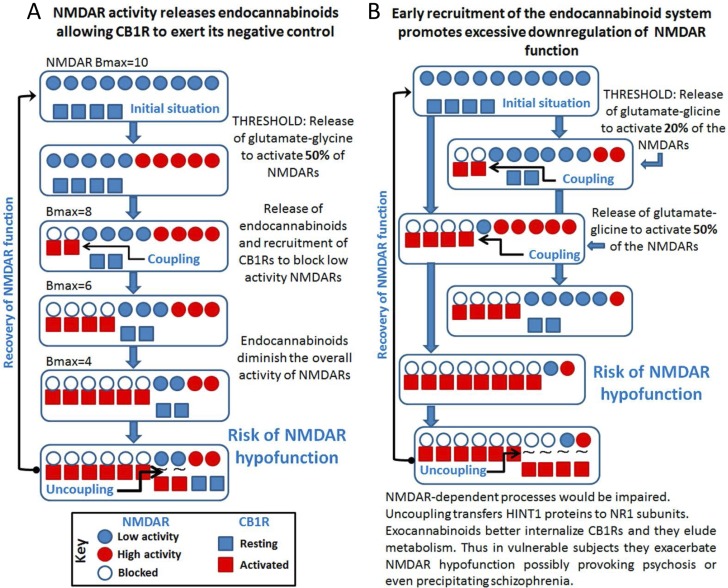
Cross-regulation between excitatory glutamate NMDAR signaling and the endocannabinoid system: Implications in psychosis and schizophrenia **A.** The activity of NMDARs demands endocannabinoid control via CB1Rs. **B.** Disproportionate endocannabinoid control could cause NMDAR hypofunction.

The functional relationship between CB1Rs and silent NMDARs depends on the HINT1-σ1R switch, and it is stabilized by endocannabinoids. The σ1R is a calcium sensor [49] that associates with the CB1R-NMDAR complex and when calcium levels are reduced, σ1R antagonists release inactive NMDARs from their association with CB1Rs through the transfer of HINT1 proteins. The freed NMDAR can then be activated, preventing endocannabinoids from producing glutamate hypofunction [39] (Figure 5A). Delays in operating this molecular switch would promote NMDAR hypofunction and the persistence of such a situation could bring about symptoms of psychosis, possibly even precipitating schizophrenia. Similarly, if the endocannabinoid system applies a disproportionate negative control on NMDAR activity (i.e.: there is early recruitment of endocannabinoids and/or an increased number of functional CB1Rs), HINT1 swaps to NMDARs, disconnecting both receptors and preventing glutamatergic hypofunction. Notwithstanding, this early and inopportune recruitment of the endocannabinoid system prevents the HINT1-primed pool of NMDARs from associating with GPCR-HINT1 complexes, thereby reducing the influence of the GPCR-NMDAR system on synaptic tone (Figure 5B). These situations may be exaggerated when exocannabinoids co-internalize CB1Rs and NMDAR subunits, accelerating the onset and duration of NMDAR hypofunction. In such circumstances, antagonists of CB1Rs or an increase in endocannabinoids could counteract the negative actions of exocannabinoids.

Hence, the endocannabinoid system as a target of exocannabinoids is a candidate to produce schizophrenia by inducing NMDAR hypofunction and/or altering NMDAR-GPCR cross-regulation [3, 39], while in humans the *HINT1* and *σ1R* genes have also been implicated in schizophrenia [114, 115, 116]. Mice lacking the HINT1 protein display altered dopamine transmission that might favor drug abuse [117], or antidepressant and anxiolytic-like behaviors [118]. Notably, σ1R ligands induce antidepressant and anxiolytic-like behaviors in mice [119, 53], effects that could derive from the regulatory role of σ1Rs on the HINT1 protein in the GPCR-NMDAR complex.

#### V.2.2 Convulsive disorders - NMDAR hyperfunction

Epilepsy is a chronic disorder suffered by approximately 50 million people worldwide (WHO, Fact sheet N° 999, May 2015). It is well established that altered central inhibitory (*e.g*., γ-aminobutyric acid or GABA) and excitatory (*e.g*., glutamate) neurotransmission plays a pivotal role in the etiology of epilepsy, with excess glutamatergic transmission and the ensuing overactivation of glutamate receptors being particularly relevant to its clinical manifestations [20, 120, 121]. Many basic and clinical studies have focused over the past two decades on NMDARs, showing how blocking or suppressing NMDAR activity can prevent, and in some cases reverse, certain pathological effects associated with neurological diseases, including epilepsy [122, 123, 82].

**Figure 6:**
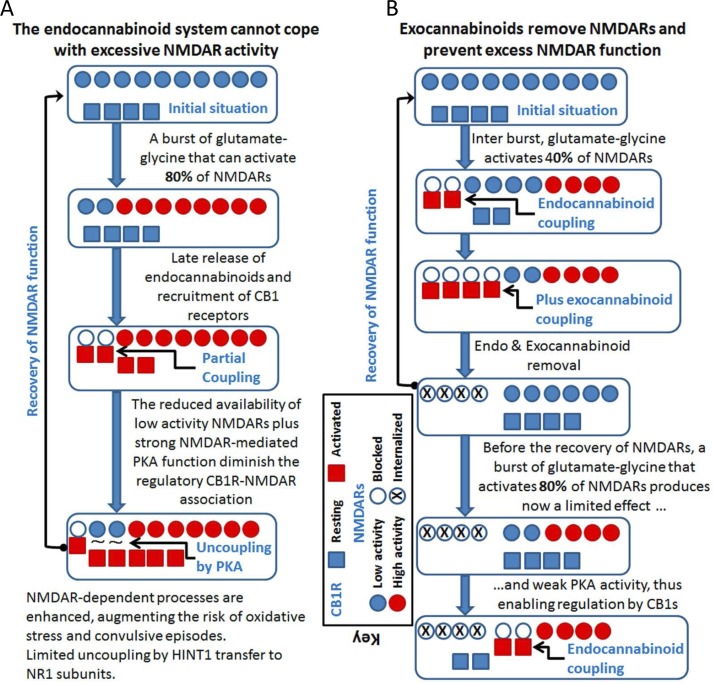
Cannabinoids can prevent the overactivation of glutamate NMDARs and reduce the incidence of convulsive episodes **A.** If the endocannabinoid system fails to control excess NMDAR activation, PKA activity increases disrupting CB1R-NMDAR complexes before they can be acted on by endocannabinoids. **B.** Exocannabinoids acting through NMDAR-recruited CB1Rs would reduce glutamate excitatory signaling. The exocannabinoids acting on the preformed CB1R-NMDAR complexes prevent PKA from disrupting these complexes (indicated as “plus exocannabinoid coupling”).

In epilepsy it would appear that NMDAR stimulation escapes from the physiological controls responsible for maintaining excitatory activity within tolerable limits (Figure 6A). Different strategies have been explored to alleviate convulsive disorders in which NMDARs are implicated. Both competitive and non-competitive NMDAR channel blockers provoke potent anti-convulsant activity [124, 125, 126], although treatment of epilepsy with chronic selective NMDAR antagonists has mostly disappointed in clinical trials [127]. The side-effects of NMDAR antagonists, pose significant problems, as they include memory dysfunction, learning deficits, psychotomimetic effects and motor disturbances [122]. High-doses of the low-affinity and non-competitive NMDAR antagonist memantine (*e.g*., 20 mg/kg) induce spontaneous motor seizures in amygdala-kindled rats [124]. Yet, at an adequate dose memantine has anticonvulsant effects against maximal electroshock seizures [128, 129] and seizures induced by different chemoconvulsants [130, 131, 126, 32]. Similarly, there is evidence that another blocker of NMDAR channels, ketamine, may also be useful to treat refractory status epilepticus [132]. Notably, these antagonists show preference for highly activated NMDARs, with the unblocked receptors functioning normally.

The drugs currently used to treat epilepsy (antiepileptic drugs -AEDs) mostly decrease electrical activity in the brain by: i) preventing neuronal depolarization by blocking excitatory sodium or calcium channels; ii) enhancing the depressor function of potassium channels; iii) inhibiting the excitatory action of glutamate; or iv) inhibiting neuronal excitability by GABA [133]. The efficacy of these medications varies in function of etiology. Despite the relatively large number of AEDs available to treat convulsive syndromes, up to 30% of patients are resistant to the pharmacotherapies currently available [134, 135, 136] and some are not candidates for surgery. Therefore, therapeutic interventions are still sought for such epilepsies unresponsive to the available treatments. Dravet and Lennox-Gastaut syndromes are examples where pharmacoresistant epilepsy not only responds poorly to conventional AEDs, but some AEDs may even worsen the patient's condition.

##### V.2.2.1 The cannabinoid system as an anti-convulsant

Intensive ongoing research with cannabinoids has produced some promising results in terms of the treatment of pediatric epilepsy and there is evidence endocannabinoid system plays a key role in regulating seizure activity in brain [137, 138, 139, 140]. NMDAR hyperactivity might be implicated in the manifestation of these convulsive syndromes and thus, interest has grown regarding the role of endocannabinoids as antiepileptic agents [141, 142]. In some preclinical models of seizures, Δ^9^-tetrahydrocannabinol (Δ^9^-THC) and synthetic CB1R agonists reduced seizure frequency or severity. However, no such effect or even potentiation of convulsive episodes has been reported elsewhere [143]. Thus, activation of CB1Rs by exogenous substances has an anticonvulsant effect in various models of experimental epilepsy, such as the maximal electroshock model of grand-mal seizure [142, 144], the rat pilocarpine model of acquired epilepsy [145, 138, 140], the *in vitro* hippocampal neuronal culture models of acquired epilepsy and status epilepticus [146, 137], the pentylenetetrazole (PTZ) model of myoclonic seizures in mice [147, 148], and the penicillin-induced model of epileptiform activity in rats [149]. Since exogenous activators of CB1Rs alleviate these epileptogenic syndromes, the endogenous cannabinoid receptors must be operative but their control on NMDARs is overridden by glutamatergic dysfunction (Figure 6B).

##### V.2.2.2 Cannabis sativa

For thousands of years, humans have used the *Cannabis sativa* plant for its sedative/hypnotic, antidepressant, analgesic, anticonvulsant, antiemetic, anti-inflammatory, anti-spasmodic and appetite-stimulating effects [86]. Thus, it is not surprising that current medicine should take advantage of the anti-epileptic potential of cannabis [150]. However, the use of cannabis by individuals to treat their epilepsy may precipitate a re-emergence of convulsive seizures when it is no longer used, while resuming cannabis consumption again controls epilepsy. Therefore, it has been complicated to obtain consistent data regarding the benefits of cannabis consumption as an anticonvulsant. In a recent informal interview of >215 patients with active epilepsy who have used recreational cannabis intermittently or regularly, more than 90% of them failed to appreciate any benefits of cannabis in seizure control. Only 7% believed that their seizures were better controlled while the remainder felt that their seizures were worse due to cannabis use [151]. In a 1976 study, 29% of patients with epilepsy reported self-medication with cannabis for their condition, of whom one reported that cannabis provoked seizures and another patient indicated an improvement with cannabis use [152]. In a more recent Canadian study, 28/165 patients with epilepsy were active users of cannabis, of whom 68% reported improvements in seizure severity and 54% in seizure frequency [153]. A careful analysis showed that cannabis use in men (but not in women) protected against new-onset unprovoked seizures and against new-onset provoked seizures when used within 90 days of seizure presentation. Yet overall, there is insufficient epidemiological data to reach hard conclusions [154], even though cannabis may protect patients from new-onset seizures and it may help patients with established epilepsy control their seizures.

#### V.2.3 Phytocannabinoids in schizophrenia and convulsive disorders

Cannabis contains several substances with unknown effects on psychosis/schizophrenia and epilepsy, including phytocannabinoids and non-cannabinoid compounds. Indeed, this plant has a complex mixture of chemicals that includes phytocannabinoids, terpenoids, flavonoids, steroids and enzymes [155] with Δ^9^-THC, cannabidiol (CBD), and cannabinol (CBN) constituting the major cannabinoids in marijuana. Despite the fact that the potential benefits remains unclear, interest in the therapeutic potential of compounds derived from *Cannabis sativa* has resurged in recent years. Thus, well-designed retrospective and prospective studies should be carried out to investigate the various cannabis preparations, strengths and compositions that have been studied. The principal psychoactive component of marijuana is Δ^9^-THC, which is a partial agonist of the CB1Rs that are primarily located in the brain (on inhibitory GABAergic and excitatory glutamatergic neurons) [156]. Δ^9^-THC is also a partial agonist of the CB2Rs that are mainly located on immune and hematopoietic cells, yet also to some extent on neural cells.

CBD is probably the most promising non-psychoactive anti-convulsant and anti-psychotic phytocannabinoid investigated to date. CBD has been seen to exert anti-convulsant effects in animal models and humans [157, 158, 159, 142], and it prevents some of the psychotic-like effects produced by Δ^9^-THC [160]. Such effects are promoted through mechanisms that remain unknown but that probably do not involve direct binding to the CB1R. In this respect, CBD only weakly competes with ^3^[H] CP55940 at both CB1Rs and CB2Rs, and at concentrations in the micromolar range [161, 162]. Despite its low affinity for CB receptors, CBD can produce effects at reasonably low concentrations and in fact, in the low nanomolar range CBD it alters the binding of agonists to the CB1/CB2 receptors [162]. However, while the role of CBD at CBRs remains controversial, its influence on endocannabinoid signaling appears convincing. Indeed, CBD potentiates such signaling, increasing anandamide levels by inhibiting its reuptake and degradation, the latter involving a dampening of FAAH expression and activity (fatty acid amide hydrolase - the enzyme involved in anandamide breakdown) [163, 164]. However, the concentrations of CBD required to inhibit anandamide reuptake and hydrolysis are quite high (>20 μM) [163].

It should be noted that CBD exerts other effects that could also contribute to its antiepileptic/antipsychotic activities. These include the modulation of the equilibrative nucleoside transporter, the orphan G-protein-coupled receptor 55 and the transient receptor potential of melastatin type 8 channel [165]. CBD modifies intracellular calcium concentrations and it inhibits T-type calcium channels [166]. At higher concentrations, CBD activates the nuclear peroxisome proliferator-activated receptor-γ (PPAR-γ) and the transient receptor potential of vanilloid type 1 (TRPV1) and TRPV2 channels [167]. In addition, CBD has anti-apoptotic, neuroprotective, and anti-inflammatory effects [168]. CBD also displays some agonist activity at α3 and α1 glycine receptors and at the transient receptor potential of ankyrin type 1 [169]. At present, there is no convincing information as to the precise molecular mechanisms by which CBD produces its antipsychotic or anticonvulsant effects. Thus, the relationship between CBD and NMDARs is apparently indirect, and it probably resides in the context of its influence on CB1Rs. Since our aim is to analyze these neural dysfunctions from a new perspective, that of the control exerted by the CB1R *via* HINT1/σ1R on NMDAR function, we will describe just a few promising findings regarding the effects of CBD on schizophrenia, and the effects of CBD combined with cannabinoids in the treatment of NMDAR convulsive episodes.

In healthy volunteers CBD attenuates the impairment of time production tasks and the euphoria induced by Δ^9^-THC [170, 171]. In humans, CBD significantly reduces psychotic symptoms in acute schizophrenia with potency similar to that of the antipsychotic amisulpride [167, 8, 172]. Notably, schizophrenic patients treated with CBD present higher anandamide serum levels than those receiving amisulpride, and in the CBD group there was a significant association between anandamide levels and improvement of psychotic symptoms [167]. Indeed, CBD inhibits FAAH activity at a concentration that does not interact with receptors commonly associated with schizophrenia, such as dopamine, GABA, serotonin and glutamate receptors. CBD appears to have pharmacological profile similar to that of atypical antipsychotic drugs in preventing human experimental psychosis, and it is apparently effective in open case reports and clinical trials in patients with schizophrenia [173]. Several mechanisms were proposed to explain the antipsychotic properties of CBD, such as the activation of CB1Rs *via* increased levels of anandamide or the activation of TRPV1 channels facilitating the pre-synaptic release of glutamate that would counteract NMDAR hypofunction. Facilitation of CB1R-mediated neurotransmission by CBD increases adult hippocampal neurogenesis, a mechanism that could improve the cognitive deficits seen in schizophrenic patients. Amongst these possible mechanisms, CBD facilitation of 5-HT1A mediated neurotransmission could account for its anti-psychotic. Besides the CB1R, certain serotonin receptors are also negatively coupled to NMDAR activity [4] and CBD displays some affinity for 5-HT1A receptors [169], providing another possible explanation for its effects that is worthy of consideration.

Alternatively, drugs like fenfluramine that acts on 5HT1R [174] can control seizures in patients with Dravet syndrome [175]. Phase 3 clinical trials with ZX008 are currently ongoing in the US and Europe (Zogenix: low-dose fenfluramine-orphan drug designation granted for ZX008 in Dravet syndrome by FDA). Therefore, it is feasible that continuous seizures (status epilepticus) can be controlled by acting on GPCRs like CB1R or 5HT1A receptors, with the HINT1-σ1R tandem coupling their function to that of over activated NMDARs. There are conflicting reports on the possible role of neurosteroids in convulsive disorders [176, 177] although the pro-convulsant effects of progesterone appear not to be related to σ1Rs given that σ1R ligands have mostly anti-convulsant effects [178, 179]. As such, recent data on the involvement of σ1R in rare CNS diseases highlights the potential of the ANAVEX 2-73 sigma ligand to treat other CNS disorders, including epilepsy [180]. Additional studies with highly selective σ1R ligands would definitely shed some light on their therapeutic potential as anti-convulsive agents.

Many of the pharmacological activities of CBD have only been established *in vivo* and hence, some of them may be due to CBD metabolites. Like most cannabinoids, CBD is metabolized extensively by the liver, where it is hydroxylated to 7-hydroxyl-CBD by cytochrome P450 (CYP) enzymes, predominantly isozymes of the CYP3A (2/4) and CYP2C (8/9/19) families [181]. These metabolites then undergo further metabolism in the liver, and their products are excreted in the feces and secreted in the urine [182]. Recent *in vitro* studies show that CBD is a potent inhibitor of multiple cytochrome P450 enzymes including CYP1A2, CYP2B6, CYP2C9, CYP2D6 and CYP3A4 [183, 184, 185, 186]. Consequently, CBD metabolism could influence the pharmacokinetics of other pharmacological agents, although there is currently little data available in this regard. In some studies, CBD has been shown to mildly augment the levels of Δ^9^-THC (metabolized by CYP2C9, CYP2C19 and CYP3A4) by reducing its conversion to 11-hydroxy-THC [187, 188]. Moreover, CBD reduces the potency of some anti-convulsants and it enhances that of others, even though it is uncertain whether this effect is a pharmacokinetic activity [189, 190].

#### V.2.4 Clinical studies

Early clinical studies to evaluate the possible efficacy of CBD and other cannabinoids in epilepsy had important methodological limitations. A recent Cochrane review identified four studies published between 1978 and 1990 that were randomized, controlled trials, blind (single or double) or unblind, and that included 9 to 15 patients [191]. These studies failed to provide evidence of cannabinoid efficacy in treating epilepsy and the main conclusion was that short term CBD treatment (in the 200-300 mg/day range) is usually well tolerated in adults [192, 158, 193, 194]. In another study, an improvement was reported in children with grand mal epilepsy that were administered isomeric homologues of TCH [195].

The use of cannabinoids in children with refractory epilepsy was assessed recently by surveying an internet-based (Facebook) group of approximately 150 children with various types of medication-resistant epilepsies, including Dravet and Lennox-Gastaut syndromes [196]. In 19 cases, 12 of whom had Dravet syndrome, parents had explored the use of CBD enriched cannabis to manage pediatric treatment-resistant epilepsy. Of the parents surveyed, 53% reported a >80% reduction in seizure frequency, with 11% of children remaining seizure free during a 3-month period. The parents also often reported better mood, improved alertness and better sleep, with no severe side effects. Interestingly, orphan drug designation has been granted by the FDA to treat Dravet and Lennox-Gastaut syndromes with the low-THC/high-CBD product Epidiolex^(R)^ (GW Pharmaceuticals). Evidence for its efficacy has come from the first pivotal Phase 3 study on Dravet syndrome where Epidiolex was compared to a placebo, measuring the frequency of convulsive seizures during the 14-week treatment relative to the 4-week basal observation period (http://www.gwpharm.com/news.aspx; March 2016). Patients receiving Epidiolex achieved a highly significant 39% median monthly reduction in convulsive seizures compared with a 13% reduction in those receiving a placebo (*p*=0.01). This difference between Epidiolex and the placebo emerged during the first month of treatment and was sustained throughout the entire treatment period. Sensitivity analyses of this primary endpoint confirmed the robustness of this result.

The complex composition of the *Cannabis sativa* plant itself makes it a challenge to understand why it apparently has contradictory effects in epilepsy. It has 489 known constituents [155] only 70 of which are cannabinoids, with the remainder including potentially neuroactive substances such as terpenes, hydrocarbons, ketones, aldehydes and other small hydrophobic compounds capable of crossing the blood-brain barrier. Strain-specific variability in the ratio of the most common cannabinoid, THC, and the second most common cannabinoid, CBD, offers further complexity when using whole cannabis as an antiepileptic agent, although most marijuana strains used to treat epilepsy are thought to have a high CBD/THC ratio. The extraction method is also critical, as the conditions and solvents used to separate these phytocompounds may alter them.

There are multiple potential routes of administration for CBD/THC. The inhaled route is the most common delivery form as a constituent of smoked cannabis used for recreational or medicinal purposes. Delivery through aerosols or vaporization using specialized devices has been examined, reporting rapid peak plasma concentrations (<10 min) and bioavailability of ~31% [197]. Combinations of low THC/high CBD have been delivered orally in an oil-based capsule in some human trials, although poor water solubility and erratic gastrointestinal absorption leads to variable pharmacokinetics. Bioavailability from oral delivery has been estimated at 6% due to significant first-pass metabolism in the liver [198]. Oral-mucosal/sublingual delivery through sprays/lozenges has similar bioavailability to the oral route but less variable (Guy and Robson, 2004: A Phase I, Open Label, Four-Way Crossover Study to Compare the Pharmacokinetic Profiles of a Single Dose of 20 mg of a Cannabis Based Medicine Extract -CBME- GWPK0112). Transdermal approaches have also been investigated but due to the strong lipophilicity, special ethosomal delivery systems are needed to prevent drug accumulation in the skin, making this approach impractical and costly [199]. The bioavailability of oral and smoked cannabis in humans was found to be around 6% and 31%, respectively, further support for a substantial first-pass effect [200, 181, 197, 201]. The adverse effects of cannabis are likely to be minimized by using active principles rather than the whole plant in medicine. Moreover, the therapeutic bioavailability of these active principles can be controlled using the adequate route of administration and dosage.

## VI CONCLUDING REMARKS

The glutamate NMDAR is implicated in certain neurological disorders and whilst psychosis/schizophrenia concurs with reduced NMDAR activity, these receptors are hyperactive in convulsive disorders like epilepsy. The use of agonists or antagonists of NMDARs to treat such conditions is commonly ineffective in clinical trials on humans as directly altering synaptic NMDAR transmission compromises neuronal survival [202]. Therefore, approaches that indirectly modulate NMDAR activity are currently being developed and validated clinically [12, 203]. Subjects with schizophrenia and bipolar disorder experience alterations to neuroactive steroids like pregnenolone, dehydroepiandrosterone and allopregnanolone [204]. These alterations might reinforce the HINT1-σ1R switch and rescue GPCR-induced activation of NMDARs, indicating that regulators of σ1Rs and HINT1 proteins [205, 9] could represent more reliable and effective therapies. In fact, preliminary clinical trials with pregnenolone highlight its potential to alleviate symptoms of schizophrenia [206], and synthetic σ1R antagonists have completed phase I safety and pharmacokinetic evaluations in humans [207]. Similarly, NMDAR-induced continuous seizures (status epilepticus) can be controlled by CB1R and here, CBD and regulators of HINT1-σ1R activity seem to be promising agents. Although the precise mechanism of action of CBD in this particular context remains unknown, its therapeutic potential may stem from endogenous compensatory systems, such as the endocannabinoid system. Research in this field is particularly relevant to treat severe seizures in pediatric epilepsy. Thus, palliative treatments for psychosis/schizophrenia and convulsive syndromes that directly focus on NMDAR or GPCR activity could be complemented or even substituted with others that modify the GPCR/NMDAR interactions.
